# Why cuckoos remove host eggs: Biting eggs facilitates faster parasitic egg‐laying

**DOI:** 10.1002/ece3.10762

**Published:** 2023-12-13

**Authors:** Longwu Wang, Huahua Zhao, Hanlin Yan, William E. Feeney, Wei Liang

**Affiliations:** ^1^ School of Life Sciences Guizhou Normal University Guiyang China; ^2^ Ministry of Education Key Laboratory for Ecology of Tropical Islands, Key Laboratory of Tropical Animal and Plant Ecology of Hainan Province, College of Life Sciences Hainan Normal University Haikou China; ^3^ Doñana Biological Station (CSIC) Sevilla Spain; ^4^ Department of Biosciences Durham University Durham UK

**Keywords:** brood parasitism, common cuckoo, egg‐laying, egg removal, help delivery hypothesis

## Abstract

Brood parasitism by cuckoos relies on manipulating hosts to raise their offspring and has evolved stunning adaptations to aid in their deception. The fact that cuckoos usually but not always, remove one or two host eggs while laying their eggs has been a longstanding focus of intensive research. However, the benefit of this behavior remains elusive. Moreover, the recently proposed help delivery hypothesis, predicting that egg removal by cuckoos may decrease the egg‐laying duration in the parasitism process caused by biting action, lacks experimental verification. Therefore, in this study, we examined the effects of egg removal/biting on the egg‐laying speed in the common cuckoo (*Cuculus canorus*) to experimentally test this hypothesis. We compared the duration of cuckoo egg‐laying in empty nests, nests with host eggs, and nests with artificial blue stick models to test whether cuckoos biting an egg/stick can significantly hasten the egg‐laying speed than no biting action. Our results showed that biting an egg or an object is associated with cuckoos laying approximately 37% faster than when they do not bite an egg or an object. This study provides the first experimental evidence for the help delivery hypothesis and demonstrates that when cuckoos bite eggs or other objects in the nest, they lay eggs more quickly and thereby avoid suffering the hosts' injurious attack.

## Graphical abstract

We showed that biting an egg or an object is associated with cuckoos laying approximately 37% faster than when they do not bite an egg or an object. This study provides the first experimental evidence for the help delivery hypothesis and demonstrates that when cuckoos bite eggs or other objects in the nest, they lay eggs more quickly and thereby avoid suffering the hosts' injurious attack.

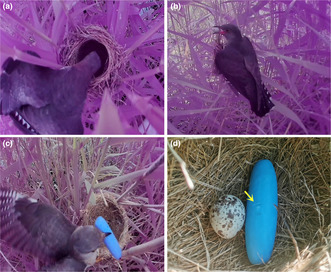

## 1 INTRODUCTION

Obligate avian brood parasites, such as parasitic cuckoos (Cuculidae) and cowbirds (Icteridae), transfer parent efforts and breeding costs to a host bird to achieve reproductive success (Davies, 2000; Soler, 2017). Because hosts suffer a huge cost of parasitism, they attempt to prevent the approach of parasites through various mechanisms, such as mobbing or attack (Feeney et al., 2012; Wang et al., 2023; Wang, Ma, et al., 2021; Welbergen & Davies, 2009). These resistance behaviors can lead to injury and even death of parasites (Gloag et al., 2013; Grim, 2005; Šulc et al., 2020; Trnka & Prokop, 2012; Zhao et al., 2022). Therefore, avoidance of host attacks and rapid, undetected egg‐laying are important adaptations in parasites (Cossa et al., 2022; Davies, 2000; Davies & Brooke, 1988; Sealy et al., 1995; Thorogood & Davies, 2016).

The egg‐laying process in parasites is extremely fast and usually only lasts 3–10 s (Davies & Brooke, 1988; Jelínek et al., 2021; Moksnes et al., 2000; Wang, Zhong, et al., 2020), compared to 20–103 min in other nonparasitic birds (Gill, 2003; McMaster et al., 2004; Sealy et al., 1995). Despite the risk of injury by the host after the parasite lays eggs, it sometimes removes one or two eggs from the host nest (Jelínek et al., 2021; Moksnes et al., 2000). This behavior has puzzled researchers, and various hypotheses have been proposed, such as “the host deception hypothesis”, which predicts that the hosts can count and would easily notice an extra egg in their nest (Hamilton & Orians, 1965), and the “help to the parasitic chick hypothesis”, which states that the female can lower energy expenditure of the parasitic chick by reducing host clutch size (Blankespoor et al., 1982; Soler & Martínez, 2000). However, most of these explanations do not sufficiently justify the egg‐pecking behavior (summarized by Šulc et al., 2016).

With the development and popularization of high‐definition photography, researchers have recorded and accumulated videos of cuckoo egg‐laying behavior. Wang, Zhong, et al. (2020) observed 53 videos of the common cuckoo (*Cuculus canorus*) (hereafter referred to as cuckoo) parasitizing the Oriental reed warbler (*Acrocephalus orientalis*) (ORW). They noted that cuckoos peck host eggs while laying their eggs. After laying eggs, some cuckoos removed the eggs that were bitten, whereas others dropped the eggs and flew away. Based on these observations, they proposed a new explanation for egg‐removal behavior, termed the help delivery hypothesis, which predicts that egg pecking and biting by cuckoos can increase the egg‐laying speed, thereby reducing the host attack probability (Wang, Zhong, et al., 2020). They emphasized that egg removal itself is simply a by‐product of egg‐laying, whereas egg pecking and biting are essential parts of its parasitism behaviors for successful egg‐laying in cuckoos.

In Europe, Jelínek et al. (2021) analyzed videos of the cuckoo parasitizing the great reed warbler (*Acrocephalus arundinaceus*; GRW) and observed that all cuckoos show egg‐searching and egg‐removal behaviors when they parasitize their hosts. After laying the eggs, some cuckoos did not remove the eggs from their mouths. They proposed that egg removal is not costly for the cuckoo and is an important parasitism strategy. Similar observations were reported in an earlier study (Moksnes et al., 2000), in which a cuckoo ate two host eggs before laying eggs, grasped a third host egg, laid her egg, and finally disappeared without removing the third host egg. In summary, the observed cuckoo behavior of biting and occasionally removing host eggs most likely facilitates faster egg‐laying, thereby limiting potential attacks by the host.

**FIGURE 1:**
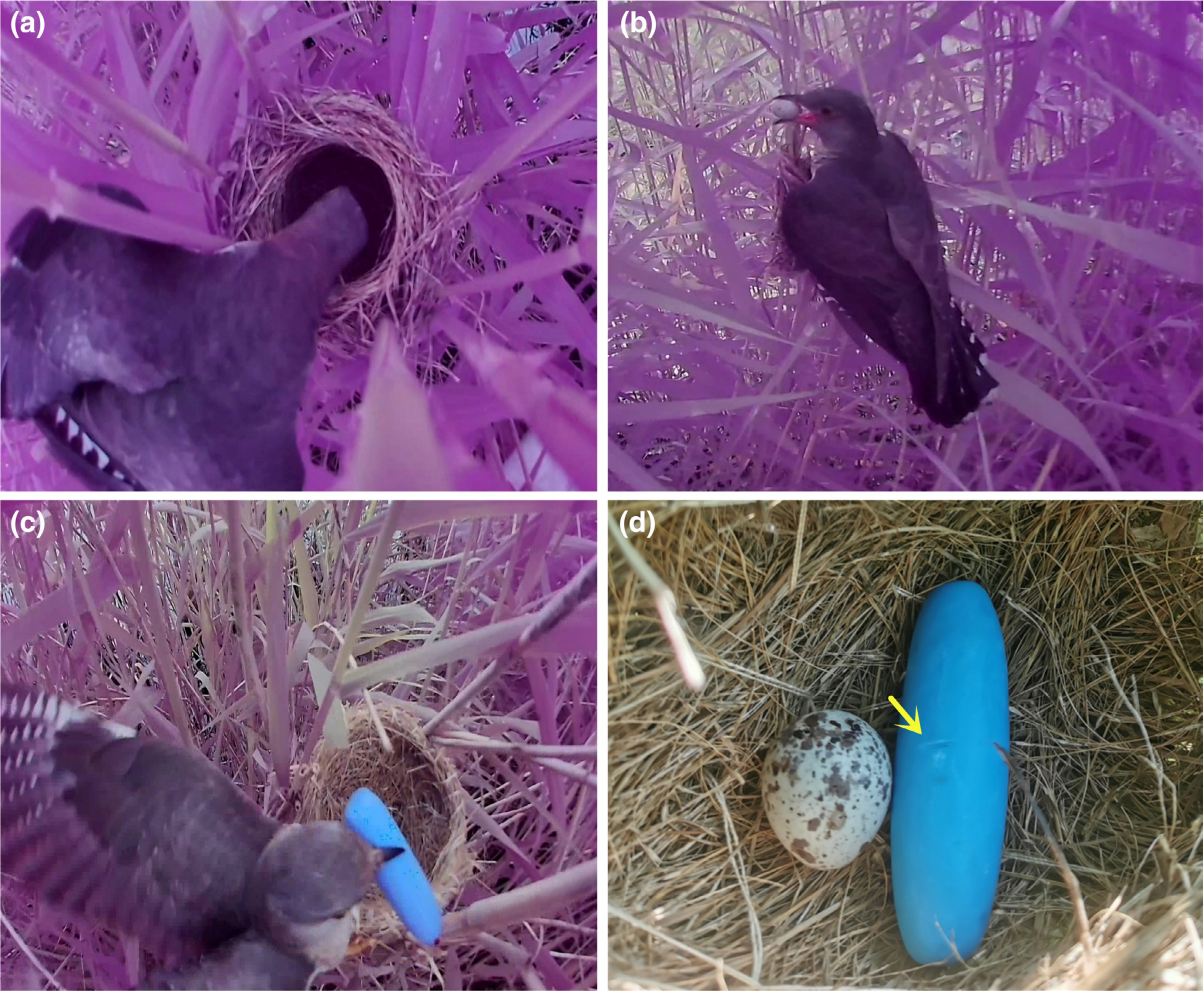
Schematic diagram of the three experiments. (a) One female cuckoo laying its egg in an empty nest, (b) One female cuckoo laying its egg in a nest with host eggs, (c) One female cuckoo laying its egg in a nest with a blue stick model, and (d) The biting mark made by one female cuckoo during egg‐laying).

In this study, the egg‐laying duration by common cuckoos while parasitizing ORW nests with different egg contents was examined using field videos to evaluate the help delivery hypothesis. The cuckoo typically removes 1–2 host eggs before laying eggs (Davies, 2000; Jelínek et al., 2021). However, when the nest is empty (0 eggs), that is, when there is no egg for cuckoos to bite, it is not clear whether the duration of egg‐laying increases, as predicted by the help delivery hypothesis. Accordingly, we recorded the egg‐laying process in empty nests (Figure 1a) and nests with eggs (Figure 1b) to compare the egg‐laying durations. Additionally, because biting is predicted to help cuckoos lay eggs, we set up an artificial blue stick as a model egg in empty nests (Figure 1c). It was hypothesized that if a cuckoo bites the blue stick before laying eggs, leaving a bite mark (Figure 1d), the action of biting facilitates egg‐laying, irrespective of the object type (i.e., stick or egg).

## 2 MATERIALS AND METHODS

### 2.1 Study area and species

The study was performed at the Zhalong National Nature Reserve (47°19′ N, 124°22′ E) in Heilongjiang and at the Sifangtuozi farm (46°12′ N, 123°84′ E) in Jilin, North China, located 110 km apart. Field experiments were conducted during the breeding season (June–August) in 2021. The two sites are both reed wetlands; the ORW breeds in reeds, and the only parasite is the cuckoo. ORW nests in the Zhalong area have a high cuckoo parasitism rate, varying from 34.3% to 65.5% over the years (Yang et al., 2017). The parasitism rate at Sifangtuozi farm was 57.5% (61/106) in 2021.

### 2.2 Field experiments and video recordings

A systematic search for ORW nests in reed swamps was simultaneously performed in these two areas. Each ORW nest discovered in the field was numbered, and the date, clutch size, location, and cuckoo parasitism status were recorded. For each new nest, a mini electronic camera (Uniscom‐T71, 70 × 26 × 12 mm; Mymahdi Technology Co. Ltd., Shenzhen, China) was used to capture cuckoo behaviors. To extend the battery life, an external power supply (20,000 mAh), sufficient to record for an entire day, was used. The recording usually started at 8:00 am, and the equipment was returned before the sun was set at 20:00 pm. We have previously found that installing video equipment does not affect ORW reproduction (Wang, Yang, et al., 2020); accordingly, the camera was set up within 1.0 m of nests to ensure that the egg‐laying process was recorded clearly. The camera was camouflaged with reed leaves and wrapped with a plastic wrap layer to prevent damage by rain (although the instrument was waterproof).

The experiment was divided into three parts to compare the duration of cuckoo egg‐laying and determine the effect of egg pecking/biting on the egg‐laying duration. In Experiment A, the duration of egg‐laying by cuckoos at new empty nests (Figure 1a, 0 eggs), in which the host had not yet laid eggs, was recorded. Additionally, from the video, it was possible to observe whether the cuckoo bit the nest material at the bottom of the nest when there were no eggs. In Experiment B, cuckoo parasitism and egg‐laying were recorded when there were 1–5 eggs in the host nest (Figure 1b); the duration of egg‐laying and egg‐biting behavior were obtained from the video. In Experiment C, an artificial blue stick model egg was used to attract cuckoo egg‐laying (Figure 1c). To determine the necessity of the egg‐biting action during the egg‐laying process, a blue stick was used as a mock host egg (70.91 ± 0.39 mm in length, 20.68 ± 0.40 mm in diameter, 18.96 ± 0.32 g in mass, *n* = 15), which was placed in a large artificial nest (116.35 ± 0.77 mm in diameter, 63.60 ± 0.57 mm in height, *n* = 15), and the nest was placed 0.5 m from an active host nest. The nest was positioned higher than the active host nest and prominently placed to ensure that when the cuckoo arrived, it would see this nest first and choose the artificial nest for parasitism (Yang et al., 2016, 2017). In this case, we observed whether the cuckoo removed or bit the blue stick. Additionally, the blue stick was relatively soft and convenient for checking cuckoos' biting marks; when the stick was not removed, bite marks were examined to establish whether the action of biting the stick promotes faster egg‐laying.

When checking nests in the field, if parasitism occurred, the video was brought to the laboratory for analysis of egg‐laying behavior. Each experimental nest was recorded for 6 days until the ORW laid a full nest of eggs, usually 4–6 eggs (Liang et al., 2014; Wang, He, et al., 2021; Wang, Zhong, et al., 2020). Preliminary studies have shown that cuckoo parasitism usually occurs during the egg‐laying period, especially when there are 1–2 eggs (Wang, Yang, et al., 2020; Wang, Zhong, et al., 2020; Yang et al., 2016, 2017), and video recording was stopped during the incubation stage.

### 2.3 Statistical analysis of the recordings

Video analysis software (Aijianji; resolution: 0.04 s per frame; Wangxu Technology Co. Ltd., China) was used to analyze the egg‐laying process and extract all relevant data, including the duration of egg‐laying (s), date, location, clutch size, host presence/absence, and whether the host eggs or the novel object were removed. To investigate whether there was a significant effect of nest content and host presence/absence on cuckoo laying time, generalized linear mixed models (GLMMs) and log‐link function were used to compare differences in the duration of egg‐laying by cuckoos between the three types of nests (0 eggs, 1–5 eggs, and blue stick model egg). For all GLMMs, video IDs were treated as random terms, and all other predicted variables were treated as fixed effects. To test whether the removal of the novel object affected the cuckoo laying speed, we used analysis of variance. Only the novel object trials were analyzed for this part of the study, as the cuckoo removed an egg from all trials in which the nest contained eggs, whereas the novel object was not always removed (removed in 4 of 12 trials) but was always bitten by the cuckoo.

All analyses were conducted using RStudio (version 2022.02.1) (R Core Team, 2022). We used the {lme4} package for all mixed models (Bates et al., 2015), model assumptions were examined using the {DHARMa} package (Florian, 2003), and pairwise tests were conducted using the {emmeans} package (Russell, 2021). The GLMM statistical analysis was conducted using IBM SPSS (version 27.0; IBM Corp., Armonk, NY, USA). Values are presented as mean ± standard deviation (SD) unless stated otherwise. Differences were considered statistically significant at the level of 0.05.

## 3 RESULTS

In the three experimental groups, videos of nests containing 0 eggs (*n* = 16 nests) (Videos S1–S16), 1–5 eggs (*n* = 19 nests) (Videos S17–S35), and the blue stick (*n* = 16 nests) at the moment of the cuckoo parasitism were recorded in the field. For the blue stick nests, the videos for 12 nests were valid (Videos S36–S47), whereas videos for 4 nests were incomplete owing to errors in the video equipment. We recorded whether the cuckoo took away the blue stick or bite marks were observed (Figure 1d). However, these nests were excluded from the analysis of egg‐laying duration due to lack of the video detail. The GLMMs indicated that clutch size, date, and location did not affect the duration of egg‐laying in cuckoos (Table 1).

**TABLE 1:** Generalized linear mixed model analyses of the effect of the nest content, clutch size, date, location, and host activity on the egg‐laying duration by female cuckoos.

Source	*F*	df_1_	df_2_	*p*
Nest content	6.491	2	40	.004
Clutch size	0.443	1	40	.51
Date	0.885	1	40	.353
Location	1.089	1	40	.303
Host activity	13.044	1	40	.01

**FIGURE 2:**
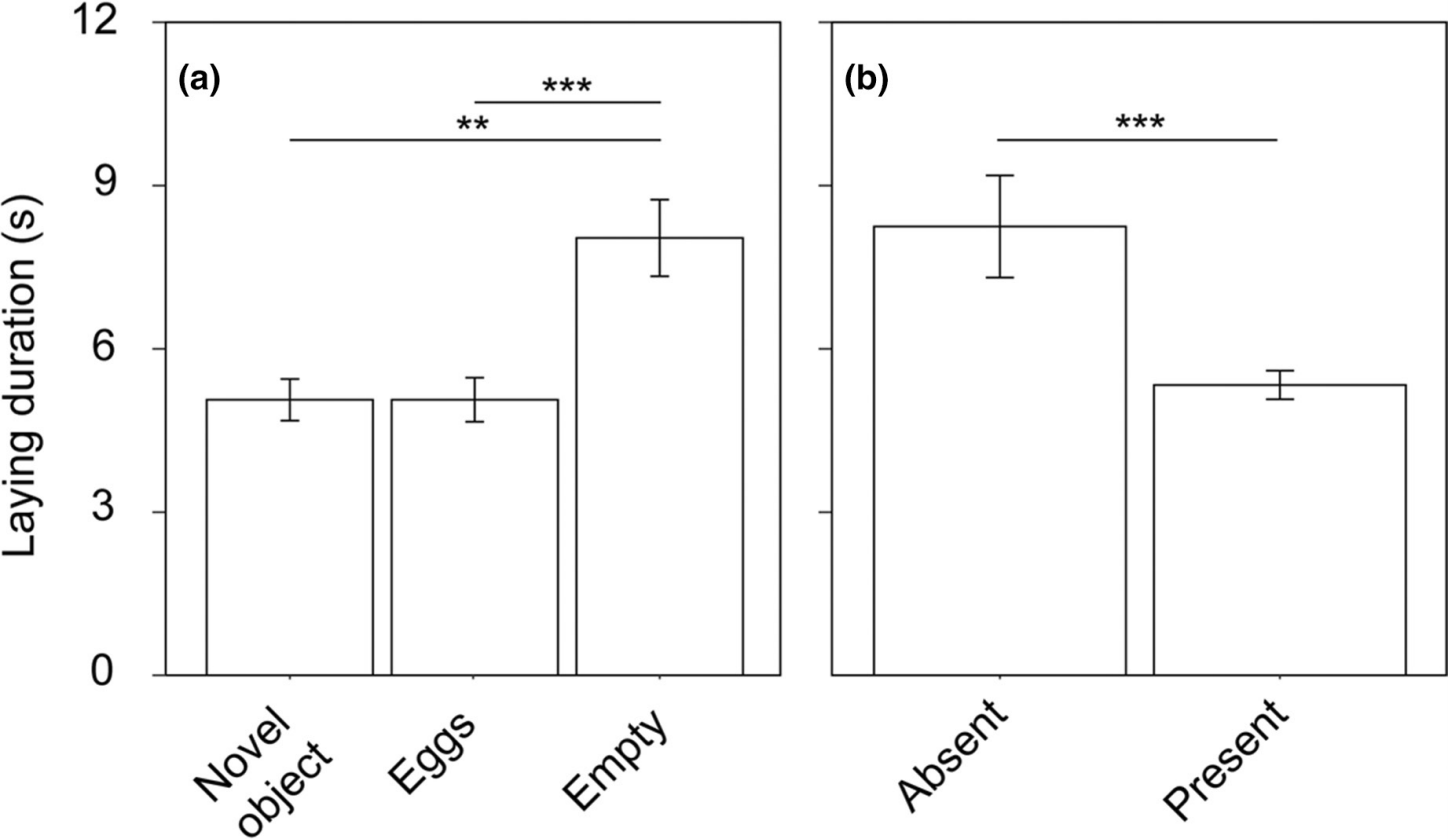
Average duration the common cuckoos spent laying their eggs in each of the three experiments (a), and the average duration the cuckoos spent laying when hosts were absent and present (b). Asterisks denote significant differences and error bars denote standard errors.

We found that nest content (0 eggs, 1–5 eggs, or novel object) had a significant effect on the egg‐laying speed (*F*_2,45_ = 10.835, *p* = .0002; Figure 2a). Particularly, laying was faster in nests containing 1–5 eggs than in empty nests (time: 8.124 s vs. 5.119 s, *t* = −4.158, df = 43, *p* = .0004) and in nests containing a novel object compared to that in empty nests (time: 8.124 s vs. 5.117 s, *t* = −3.863, df = 43, *p* = .0011); however, laying was not faster in nests containing 1–5 eggs compared to that in those containing the novel object (time: 5.119 s vs. 5.117 s, *t* = −0.149, df = 43, *p* = .988) (Figure 2a). We also found that host presence affected the egg‐laying duration by cuckoos, with cuckoos laying more quickly when the host was present than when they were absent (time: 5.336 s vs. 8.248 s) (*F*_1,44_ = 16.949, *p* = .0002; Figure 2b).

Additionally, in the blue stick experiment, when the cuckoo removed or bit the blue stick model while laying eggs, bite marks remained (Figure 1d), indicating that these actions, in which the cuckoo exerts force to bite the egg or stick, are conducive to rapid egg‐laying, thus supporting the help delivery hypothesis. Finally, we analyzed whether the removal of an object from the nest, rather than just biting an object and leaving it in the nest, affected laying speed, and found no effect (*F*_1_ = 2.76, *p* = .127), suggesting that the biting action, rather than removing an object, is associated with faster laying.

Host activity affected the duration of egg‐laying in cuckoos (*F*_1,40_ = 13.044, *p* = .01; Table 1). We further analyzed the correlation between the cuckoo egg‐laying duration and host activity in three experiments. When the host was absent, there were no obvious differences in the egg‐laying speed among nests with 0 eggs, 1–5 eggs, or a blue stick (*F*_2,10_ = 2.479, *p* = .134; Table 2), demonstrating that cuckoos can lay eggs relatively slowly when the host is absent (i.e., when attacks from hosts are less likely). However, when the host was present, the egg‐laying duration was significantly shorter in nests with eggs to bite than in empty nests (*F*_2,31_ = 11.796, *p* < .001), indicating that the threat posed by the host and the presence of eggs to bite both prompted faster egg‐laying to avoid attacks by the host.

**TABLE 2:** Correlation between the duration of cuckoo egg‐laying and host absence or presence under different nest contents.

Nest content	Host activity	
	Absent (s)	Present (s)
No egg	10.230 ± 3.453	6.860 ± 1.461
One to five host eggs	6.970 ± 2.984	4.625 ± 0.964
Blue stick model	5.986 ± 1.628	4.862 ± 1.188
*F*	2.479	11.796
df	2, 10	2, 31
*p*	.134	<.001

## 4 DISCUSSION

Our results showed that cuckoos that bite an object when they lay their eggs do so significantly faster (~37%) referring to duration than those that do not bite any object. The increase in laying speed occurs regardless of whether the bitten object is a host egg or a novel object (5.117 s and 5.119 s, respectively, compared with 8.124 s), suggesting that it is the biting process that enables faster laying. Similar to previous studies (Jelínek et al., 2021), we found that cuckoos were more likely to lay quickly when hosts were present, highlighting that the urgency faced by the present danger of a host can hasten laying. Overall, our results indicate that the most likely explanation for cuckoos biting and/or removing a host egg during laying is that the process of biting the host egg aids in speeding up the laying process and that any benefits conferred through the removal of the egg represent secondary benefits.

Diverse taxa use external forces to accelerate childbirth or egg‐laying in nature. In this study, during the process of recording parasitism by the cuckoo, we incidentally recorded a critical event in ORW eggs laying in one nest. The female made a low‐pitched groan, and her body shook rhythmically during egg‐laying. She laid down in the nest for approximately 10 min before delivery and then stood up and began to accelerate delivery. She lowered her head with continuous low groans and shaking. This process lasted 4 and 18 s, respectively. She then sat in the nest to rest for approximately 10 min and left the nest (see Video S48). The domestic duck (*Anas platyrhynchos domesticus*) constantly flaps its tail and grips the ground with its foot during egg‐laying (Wang et al., 2018). The ostrich (*Struthio camelus*) appears anxious before egg‐laying and runs back and forth along a certain route. Finally, it will stand still and forcefully accelerate the delivery until just before egg‐laying, at which point it lays down to prevent the eggs from falling on the ground and being broken (Dong et al., 2001). In the above examples, birds used different methods to improve the success of egg‐laying. Even in human beings, pregnant women clench fists and grind their teeth or practice some special postural guidance from midwives for delivery during childbearing (Chang, 2014; He & Chen, 2015; Lundgren & Berg, 2007). Our study showed that cuckoos bite eggs or sticks and that cuckoos left bite marks on sticks when laying eggs (Figure 1d), further demonstrating that they exerted force. This biting may help the cuckoo bear efforts and difficulties of laying eggs and thereby speed up the entire egg‐laying process.

Cuckoos are at risk of attack by their hosts, with the potential for injury or death (Gloag et al., 2013; Jackson & Kyne, 2010; Zhao et al., 2022). Šulc et al. (2020) found a dead common cuckoo floating in water under a GRW nest. The cuckoo was chased and attacked by the host during parasitism and ultimately died. Another 10 cases of dead female cuckoos have been mostly found under GRW nests and in other avian host–parasite systems (reviewed by Šulc et al., 2020). For cuckoos, rapid egg‐laying and flying away in due course can reduce the risk of attacks (Davies, 2000; Soler, 2017; Wang et al., 2023). In addition to avoiding attacks, quicker egg‐laying can reduce host attention, thereby reducing the parasitic egg rejection risk (Davies, 2000).

Attacks by hosts also accelerate egg‐laying. Jelínek et al. (2021) found that cuckoos lay eggs twice as fast under attack by the GRW than in the absence of a host. They proposed that a host attack accelerates egg‐laying by parasites; in the case of a host attack, cuckoos leave immediately after laying eggs (*n* = 91), whereas when the host is absent, some cuckoos remain at the nest for a period after laying eggs. Consistent with these results, we also found that the presence of the host had a significant effect on egg‐laying by cuckoos. Additionally, videos showed that all of the laying cuckoos bite eggs. Furthermore, we identified that the two factors influencing the egg‐laying duration by cuckoos are host presence and egg biting.

Several hypotheses proposed to explain cuckoo egg removal have emphasized different standpoints (Šulc et al., 2016); however, none of these hypotheses can fully explain why the cuckoo needs to peck the host egg, and egg removal does not always occur. For example, previous hypotheses cannot explain the egg‐searching and egg‐removal behaviors of cuckoos in an empty nest (Honza et al., 2020; Jelínek et al., 2021; Wang, Yang, et al., 2020). Additionally, when a nest contains more than one egg, some cuckoos remove one egg, whereas others remove two or even three eggs (Davies, 2000; Šulc et al., 2016). Furthermore, in each case of parasitism, the cuckoo always bites eggs consistently in the nest during the laying process; however, some cuckoos may not remove any egg. Although some individuals displayed signs of urgency and pushed eggs out, most videos showed that the cuckoos deliberately dropped the eggs from their bills and then flew away. For example, in an eight‐year study of GRW, some cuckoos did not hold eggs (Šulc et al., 2016), and in ORW, this proportion was 51% (Wang, Yang, et al., 2020). Even at the nestling stage, cuckoos remove nestlings when they arrive at the nest (Honza et al., 2020; Wang, He, et al., 2021). In previous studies, given the limitations of video recordings, inferences were generally based on limited observations of cuckoo parasitism. Moreover, hypotheses proposed based on the ability of the host or the cuckoo to count eggs have neglected the key point that, in all cases of parasitism, the cuckoo bites eggs or objects in the nest; therefore, the question of why cuckoos remove eggs is irrelevant, as this is just a side effect of egg pecking and biting behavior. Future studies should focus on the generalizability of the help delivery hypothesis by analyzing different populations of cuckoos, different host species, and other parasitic species. In addition, it remained unclear as to why does the cuckoo peck an egg or an object to help them bear efforts and difficulties of laying eggs.

In summary, we verified the help delivery hypothesis by analyzing the duration of egg‐laying by cuckoos in the field. In other words, laying cuckoos need to find eggs or other objects in the nest to bite. The main function of pecking or biting eggs/objects is to increase the egg‐laying speed, accompanying this behavior to facilitate and overcome the difficulties associated with laying eggs, which ultimately speed up the laying process itself, thereby reducing the chances of encountering the host. Egg removal after egg‐laying is a side effect of egg pecking or biting, which is a prediction of the help delivery hypothesis. The help delivery hypothesis can also explain why cuckoos sometimes remove one or two host eggs but not always when they finish egg‐laying.

## AUTHOR CONTRIBUTIONS

**Longwu Wang:** Conceptualization (equal); data curation (equal); formal analysis (equal); investigation (equal); methodology (equal); writing – original draft (equal). **Huahua Zhao:** Investigation (equal). **Hanlin Yan:** Investigation (equal). **William E. Feeney:** Formal analysis (equal); software (equal); validation (equal); writing – review and editing (equal). **Wei Liang:** Conceptualization (equal); data curation (equal); funding acquisition (lead); resources (equal); supervision (lead); validation (equal); writing – review and editing (equal).

## FUNDING INFORMATION

This work was supported by the National Natural Science Foundation of China (Nos. 31960105, 32260253 to LW, 31970427, 32270526 to WL). LW was funded by the Guizhou Natural Science Foundation (No. ZK[2022]‐316), and WL supported by the specific research fund of The Innovation Platform for Academicians of Hainan Province.

## CONFLICT OF INTEREST STATEMENT

The authors declare that they have no competing interests.

## Supporting information


VideosS1‐S48

## Data Availability

Videos of cuckoo laying behavior, and data in this manuscript were submitted as Supplementary Materials and are available at the Dryad Digital Repository: https://datadryad.org/stash/share/brcGHjpX‐OZpf7gO_1dwtt01XDlTfq16qu6ez3WY2fM.
